# A multi-scale modeling approach for studying cortical lesions as a cause for epilepsy

**DOI:** 10.1186/1471-2202-12-S1-P350

**Published:** 2011-07-18

**Authors:** Sid Visser, Esther Holleman, Wilco Bouwhuis, Hil GE Meijer, Michel JAM van Putten, Stephan A van Gils

**Affiliations:** 1 Department of Applied Mathematics, University of Twente, Enschede, 7500 AE, Netherlands; 2 MIRA-Institute for Biomedical Technology and Technical Medicine, University of Twente, 7500 AE,The Netherlands; 3 Graduate School of Life Sciences, University of Utrecht, Utrecht, 3508 TC, The Netherlands; 4 Department of Applied Physics, University of Twente, Enschede, 7500 AE, The Netherlands; 5 Department of neurology and clinical neurophysiology, Medisch Spectrum Twente, Enschede,The Netherlands

## 

Traumatic brain injury (TBI) may result in post-traumatic seizures and epilepsy. Approximately 5-7% of TBI patients suffer from at least one seizure [1]. The pathophysiological mechanisms are not completely understood, and may also differ between early seizures (< 2 weeks of the injury) and the development of post-traumatic epilepsy. This includes the effects of direct physical trauma, excitotoxicity due to iron released from the blood [2] and cytokine TGF-β in blood-brain-barrier-mediated activation of astrocytes [3]. In this work we propose that a reduction in network connectivity, as presumed present in several cases of TBI, may result in seizures and epilepsy.

We use a realistic model of neocortex consisting of six different, multi-compartmental neurons and Hodgkin-Huxley like ion-channel dynamics [4]. Using physiologically realistic connectivity parameters, we analyze networks of different sizes; ranging from a microcolumn of 656 neurons to a mesocolumn that contains 20k neurons. In these networks, small lesions are introduced to simulate axonal and dendritic damage, thereby limiting action potential propagation. Furthermore, we analyze a lumped model of neocortex that is shown to correspond to the detailed model of the microcolumn [5]. This model consists of a system of two differential equations with two fixed delays. By using an automated parameter estimation method, parameters are identified for which the model's behavior closely resembles that of the realistic model. Subsequently, the dependency and sensitivity on these parameters are studied with bifurcation analysis. We generate a mesocolumn by linking several lumped units together. Lesions are then introduced by breaking or reducing some of the connections between the populations. We also study this case using both analytical and numerical bifurcation methods.

The ratio between excitatory and inhibitory connections is analytically determined as a function of network size. It is found that, compared to large networks, small networks tend to have a relatively larger number of excitatory connections than inhibitory connections. This suggests that a lesion splitting the network into smaller sub-networks, could increase the ratio of excitatory and inhibitory?connections in a particular sub-network. Choosing parameters that correspond to a region of multistability, as determined by the bifurcation analysis, enables us to create an epileptic focus that spreads epileptiform activity to neighboring areas.

## Conclusions

By using multi-scale modeling, large-scale simulations, bifurcation analysis, and parameter estimation, we study the effects of small lesions in neocortex. From the large-scale simulations we find that “neuronal peninsulas”, created by these lesions, may evolve into epileptogenic networks. By studying bifurcations of a lumped model with suitable parameters, regions of multistability are identified that are hypothesized to correspond with epilepsy.
